# Experimental and numerical simulation of solute transport in non-penetrating fractured clay

**DOI:** 10.1038/s41598-022-19117-4

**Published:** 2022-08-30

**Authors:** Jun Liu, Yue Su, Huan Shen, Yaqiang Cao, Wenjie Yang, Yong Huang

**Affiliations:** 1 Changzhou Architectural Research Institute Group Co., LTD, Changzhou, 213001 China; 2 School of Earth Sciences and Engineering, Hohai University, Nanjing, 211100 China

**Keywords:** Environmental sciences, Hydrology

## Abstract

A set of one-dimensional experimental device for solute transport in non-penetrating fractured clay are developed, which can study the laws of groundwater flow and solute transport under different hydraulic heads, fractured aperture, and thickness of non-penetrating zones. The experimental results show that the solute will quickly reach the bottom of the clay along the non-penetrating fracture, and there is an obvious dominant flow phenomenon compared with the intact clay. According to the experimental data and numerical calculation results, the model parameters of the fracture and each soil layer were identified, and the verified numerical model was used to simulate the solute transport in the non-penetrating fractured clay. The numerical results show that the increase of the thickness for the non-penetrating zone has a significant improvement on the anti-seepage ability of clay, and the increase of the hydraulic head pressure and fractured aperture leads to a faster growth rate of the solute concentration, which indicates that the solute breaks down the lower impermeable clay layer under high head pressure. The research results are of great significance for the bottom anti-seepage layer similar to landfill projects.

## Introduction

During construction or production, the compacted clay produces a variety of changes in properties, the most obvious of which is the formation of fractured clay^1–4^. Fractured clay has great irregularity and unpredictability, that is, there are fractures that run through the entire clay layer or there are non-penetrating fractures that partially penetrate the clay^5,6^. Non-penetrating fracture refers to the contact area with discrete distribution in the fracture, the rest of the fracture is open and the opening degree does not change much, and the water flow only moves in a limited channel^7–9^. The solute will quickly pass through the clay impermeable layer in the penetrating fracture, causing the solute to quickly reach the stratum at the bottom of the impermeable layer, affecting the soil and groundwater^10,11^. In the fracture with non-penetrating zone, the solute enters the impermeable layer in the form of a relatively preferential flow. At the position of the fissure, the thickness of the clay layer decreases and the water head pressure increases. In this case, the breakdown time of the clay anti-seepage layer is shortened, and the anti-seepage effect is weakened or even missing, which will cause huge pollution to the natural environment and huge economic losses to engineering projects^12–14^. With the rapid development of economy and society, the problem of groundwater contamination is receiving widespread concern worldwide. The groundwater has complex distribution, slow flow rate and poor self-purification ability, which make it more difficult to manage once pollution is caused^15–17^. The study of the migration law of groundwater contaminants in fractured clay has become the important research hotspot. The fracture network in clay layer with hydraulic connections is often the main pathway for the migration of groundwater contaminants. The existence of fractures can cause groundwater contaminants to bypass the clay matrix and move rapidly through the clay layer along the fracture channels, the preferential flow phenomenon can be observed. The groundwater contaminants will quickly reach the deep soil layer or deep groundwater aquifer, the soil and groundwater in the region will be seriously contaminated^18,19^.

Early research on solute migration in clay at home and abroad mainly existed in compacted clay, that is, the migration of solute was observed in intact clay^20–23^. During the past few decades, the research on the migration law of solute in fractured clay has been developed. At present, a large number of scholars have carried out a lot of research on the migration law of solute in fractured clay^24–26^. Lapidus and Amundson^27^ first put forward a mathematical model of solute transport, which is similar to the current general convection–dispersion equation, which opened the prelude to the study of groundwater solute transport. Tang et al.^28^ proposed the first widely used simple analytical solution for solute transport in a single fracture. Torres-Gonzalez^29^, Meiri^30^, Barton et al.^31^, and others used numerical methods to solve various groundwater problems, and first proposed the calculation methods of element finite element and element finite difference numerical models.

Laboratory experiments and numerical simulations are two commonly used methods for simulating the transport law of contaminants^32–34^. The method of laboratory experiment is to make the actual model into a small-scale model according to a certain proportion to reproduce a certain phenomenon. Numerical simulation refers to the use of computer and programming language to write software to simulate the actual hydrogeological conditions, and obtain the transport law of contaminants under the corresponding conditions^35–37^. Over the past decades, scholars have gradually applied laboratory experiments and numerical simulations to the study of the migration law of solute in fractured clay^38–40^. Zilberbrand and Gvirtzman^41^ studied groundwater solute transport under experimental conditions of large-diameter bore-holes. To study the migration properties of solute in fractured clay, tracer experiments have been performed in the laboratory by Neretniekset et al.^42^, Widestrand et al.^43^, and Cherubini et al.^44^. Heyer et al.^45^ used a homemade experimental setup to measure real-time flux in fractured media. The migration of solute in fractured media is affected by the existing preferential flow, the interaction of the three movements of fractures and matrix, adsorption and desorption, and other influences. In order to fully reflect the advantages of micromodel technology in the study of contaminant migration in fractured media. Wan and Tokunaga^46,47^ improved it so that the microfractures are the same size as fine-textured sandstone, and the model can control their boundary conditions. Seol et al.^48^ studied the two-dimensional parallel plate fracture-matrix system by numerical method. Ji et al.^49^ investigated the effect of groundwater on the Dense nonaqueous-phase liquid (DNAPL) in a discrete fractured grid, and the experimental and numerical simulations of water flow around the real DNAPL are important for its migration path, velocity and channel pattern in the fractured grid Impact. Liu et al.^50^ used a numerical simulation method to study the characteristics of water flow in a two-dimensional fracture network. Thousands of fractures were randomly generated in a large area. The simulation results show that the direction of water flow is mostly vertical, that is, it is mainly affected by gravity. Natarajan and Kumar^51^ analyzed the migration mechanism of contaminants in fractured clay porous media using numerical simulation.

However, the study of solute transport in non-penetrating fractured clay is still in the developing stage. Most of surveys and studies focus on solute transport studies in fracture media and penetrating fractured clay, and the law of solute transport in non-penetrating fractured clay is still neglected^52–54^. One of the challenging problems is how to apply laboratory experiments and numerical simulation techniques to observe and predict the migration law of solute in non-penetrating fractured clay. Understanding and studying the migration law of solute in non-penetrating fractured clay is beneficial to study the migration of solute under relatively preferential flow conditions, and can provide a scientific basis for predicting groundwater pollution and treatment. In this paper, a set of one-dimensional experimental equipment for solute transport in impermeable fractured clay was developed to study groundwater flow and solute transport laws under different hydraulic heads, fracture apertures and thickness of impermeable zones.

## Materials and methods

### Experimental model

The one-dimensional experimental model is mainly composed of four parts: tracer injection device, plexiglass cylinder, hose and peristaltic pump. The design idea is to make a platform on the fixed iron frame, fix a container as a tracer injection device, open at an appropriate position of the container and connect it with a hose. The tracer in the container is injected into the plexiglass cylinder through the hose. During the experiments, the peristaltic pump sends the tracer into the injection device. The excess tracer flows out from the fixed outlet on the injection device through the hose to ensure that the tracer in the injection device is injected into the plexiglass cylinder at a constant height. So, the tracer solution can be recycled.

**Figure 1:**
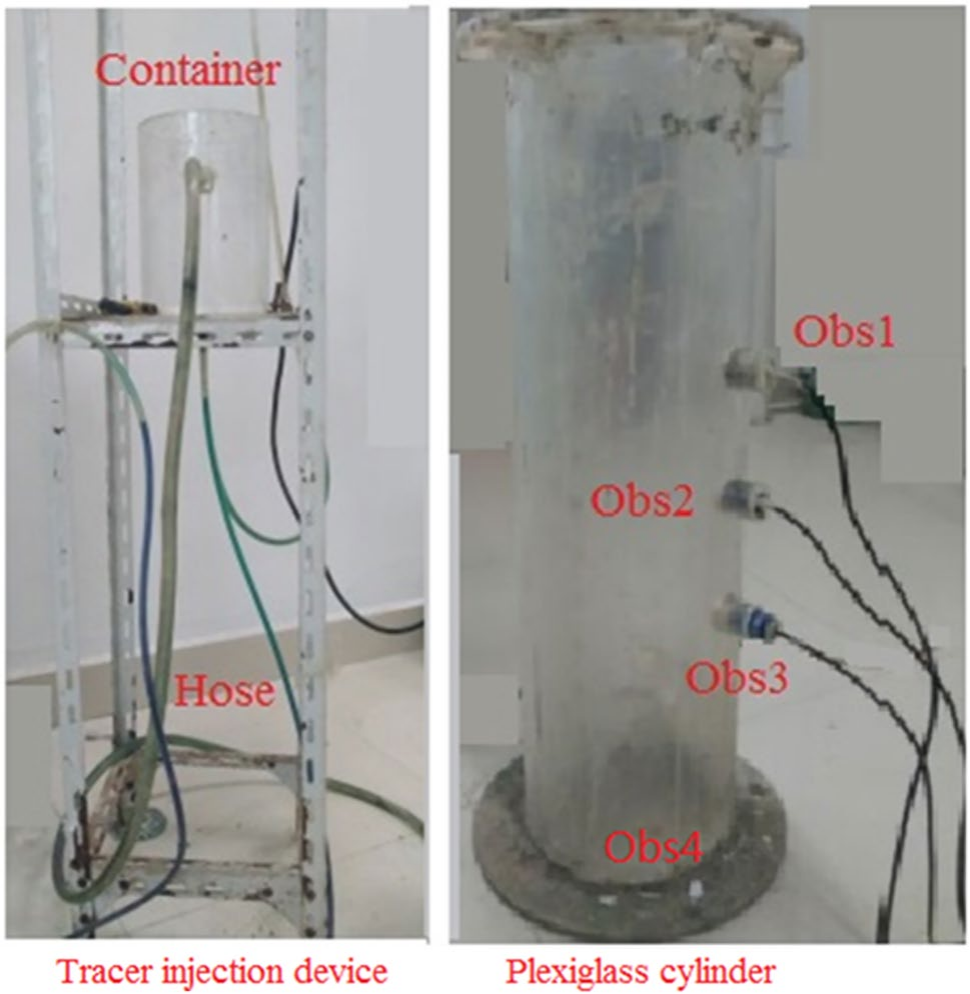
Physical map of the experimental device.

During the experiments, the tracer injection device is kept at a fixed height. Also, the height can be adjusted according to the needs of different experiment. The device is easy to disassemble and reuse. The tracer injection device can be placed on a tripod. The tripod is made of stainless-steel alloy, which is light in weight, high in strength, good in toughness, easy to process, and assembled and fixed by nuts. A fixed platform is set in the middle of the tripod, which is fixed in the hole in the frame by nuts, and the platform can move up and down. The purpose is to set different injection heights of the tracer. The interval distance is 4.7 cm (Fig. 1).

The main component of the plexiglass cylinder is polymethyl methacrylate, which is a polymer compound formed by the polymerization of methacrylate. This material has been widely used in various industries and has the advantages of high strength, corrosion resistance, and small specific gravity. It meets the needs of this experiment. The cylinder model device is made of plexiglass with a thickness of 5 mm, a height of 70 cm, and an inner diameter of 18 cm. Three observation holes of salinity sensor are installed on one side of the device, and the distances are 25 cm, 35 cm, and 45 cm from the bottom of the device, respectively. They are used to place the salt sensors with a number of FJA-10. The numbers of observation holes are Obs1, Obs2, and Obs3 (Fig. 1). An outlet is set at the bottom of the model, so that the tracer flows out after passing through the model device, and the concentration of the tracer is monitored by Obs4. The sensor probe is fixed in the cylinder, so that the probe just touches the clay in the cylinder without destroying the integrity of the clay.

### Experimental materials

#### Clay

The particle size of the clay used in the experiment is mainly concentrated between 0.075 and 0.5 mm. After analyzing the particle size of the clay sample, the percentage of different particle sizes of the soil sample is obtained (Table 1).

**Table 1:** Particle analysis statistics for clay.

Particle size (mm)	Weight of soil sample (g)	Percentage of total weight (%)	Cumulative percentage (%)
< 0.075	19.2	3.84	3.84
0.0075–0.1	85.1	17.02	20.86
0.1–0.125	39.7	7.94	28.8
0.125–0.2	95.6	19.12	47.92
0.2–0.25	120.9	24.18	72.10
0.25–0.5	75.3	15.06	87.16
0.5–1.0	60.2	12.04	99.2
1.0–2.0	4.0	0.8	100.0

#### Fractured material

**Figure 2:**
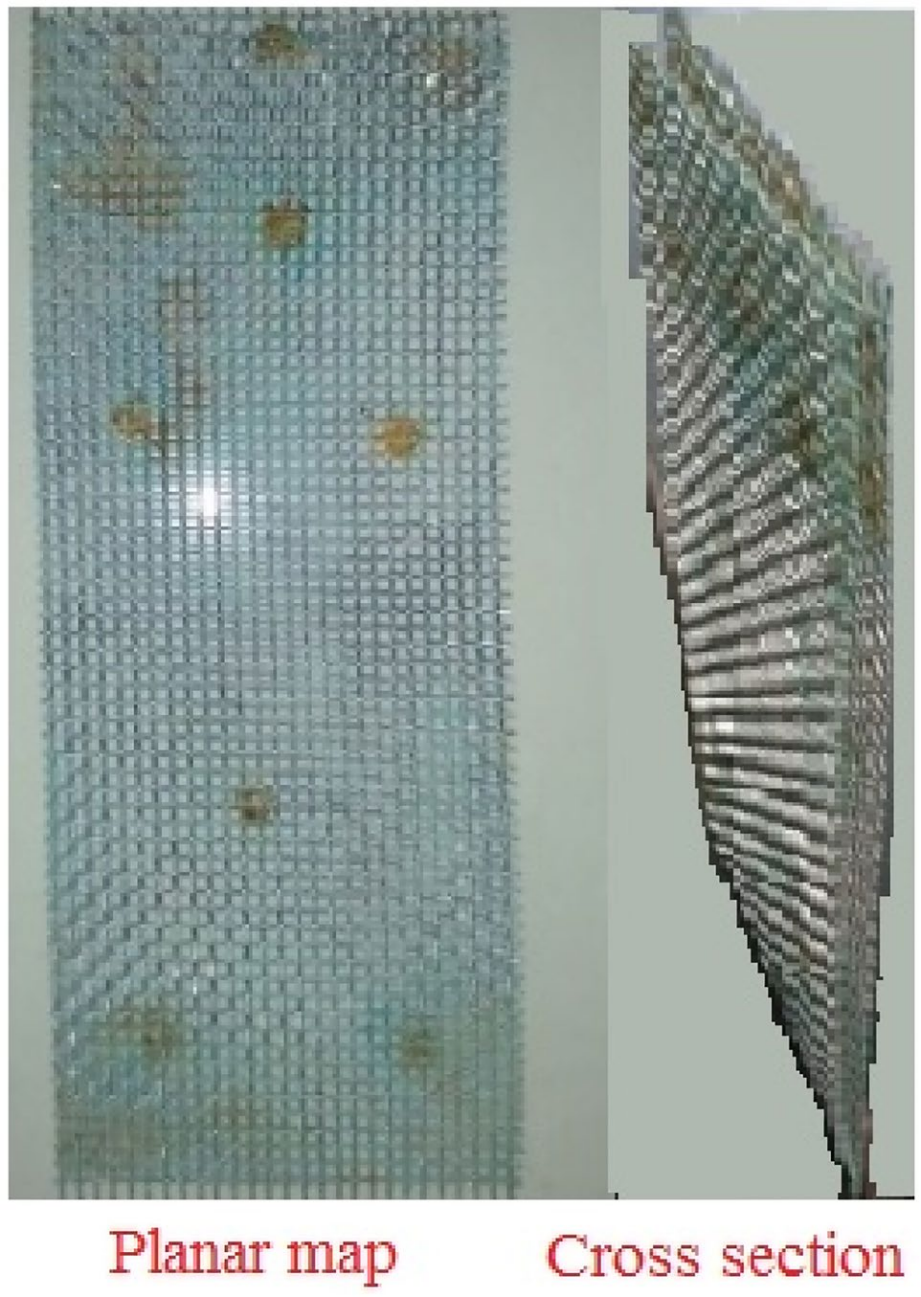
Fractured material.

The fracture used in the experiment are made of stainless-steel wire mesh, which has high hardness and is not easy to chemically react with the tracer, and is separated by a nut in the middle. A 5 mm wide fracture was set in the clay of the cylindrical model, and the length of the fracture was 25 cm, which was smaller than the length of the model device and was a non-penetrating fracture (Fig. 2). The thickness of the clay layer in the cylindrical model is 28 cm. After the fracture is placed in the clay layer, a 3 cm thick clay is set as a non-penetrating zone, and the bottom of the clay layer is coarse sand and gravel.

After the clay and fractured materials are prepared, the experiments can be performed. The clay is put into the pot, and added water to fully saturate and settle. The bottom of the cylindrical model is loaded with 7 cm thick gravel and 17 cm fine sand in sequence. A filter screen is placed between the gravel layer and the sand layer to prevent the fine sand from entering the gravel layer. A filter screen is laid on top of the sand layer. Then the clay is put into the model, let it settle naturally for 3 cm, and form a clay non-penetrating zone. The fracture is placed in the middle of the device, perpendicular to the axis of the monitoring hole. The saturated clay is filled around the fracture until the fracture is at the same height as the clay-sand soil. After filling the soil samples, let them stand still for about 5 days. It should be pointed out that when the experiment is standing still, the clay in the device will partially settle. After the settlement, it is necessary to continue to add the clay to ensure the integrity of the clay. After the clay is filled, check whether there is clay entering the fracture, and ensure that no clay enters the fracture before the test can be carried out.

#### Tracer

The tracer used in the experiment was NaCl solution, which was detected by a salt sensor. In order to display the solute migration more intuitively, a food additive bright blue was added to the NaCl solution as an indicator. The bright blue was mainly composed of benzaldehyde-o-sulfonic acid and *N*-ethyl, *N*-(3-sulfobenzyl)-aniline is obtained by condensation and oxidation. It is easily soluble in water (18.7 g/100 mL, 21 °C), and it is a greenish blue solution with strong light resistance and heat resistance. It is stable to citric acid, tartaric acid and alkali, does not react with soil samples and model materials, and is cheap and easy to obtain. In the experiment, the condition of solute transport can be preliminarily judged by the change of color. The tracer solution used in this experiment is NaCl solution with the concentration of 60 g/L, and the solvent is tap water. The experimental instruments include electronic balances, buckets, stirring rods, measuring cylinders, etc.

### Experimental measurement

**Figure 3:**
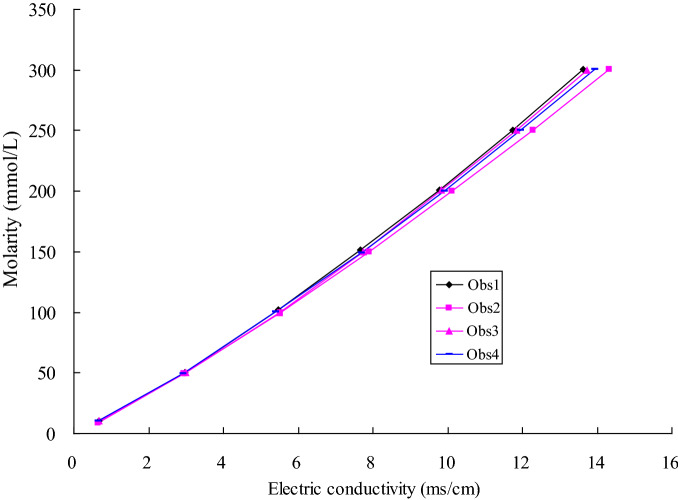
Relationship between molar concentration and electric conductivity of each salt sensor.

The content of salt can be calculated by the measurement of electric conductivity. The selected detection instruments are salt sensor with a number of FJA-10 and portable digital electric conductivity with a number of DDB-2, which is produced by Nanjing Chuandi Equipment Co., Ltd. The salt sensor has a builtin temperature sensor, which uses a graphite electrode and has the advantages of corrosion resistance and high accuracy. The portable digital conductivity has a built-in temperature compensator. The electric conductivity is measured when the salt sensor is connected to the conductivity at 25 °C. The electric conductivity value at 25 °C multiplied by the electrode constant is the measured conductivity. The salt content of clay can be obtained by the standard curve method. During the experiments, the electric conductivity of the tracer is mainly measured, and it can be converted into the solute concentration using the standard curve method, which is provided by Nanjing Chuandi Equipment Co., Ltd. The salt sensor placed at Obs1 is No. CD1800, Obs2 is No. CD1801, Obs3 is No. CD1802, and Obs4 is No. CD1803. The relationship between the solute concentration and electric conductivity is shown in Fig. 3.

The relevant parameters for converting the electric conductivity measured by each observation hole into the solute concentration are listed in Table 2. During the experiments, based on the relevant parameters in Table 2, the concentration of each observation hole can be obtained.

**Table 2:** Conversion parameters for solute concentration in each observation hole.

Measured time (days)	Observation holes	Salt sensor	Conductance (ms)	Electrode constant	electric conductivity (ms/cm)	Molarity (mmol/L)	Solute concentration (g/L)
0	Obs1	CD1800	0.45	1.70	0.77	10.769	0.63
10	Obs2	CD1801	6.96	1.71	11.90	241.38	14.12
30	Obs3	CD1802	12.62	1.71	21.58	718.46	42.03
60	Obs4	CD1803	19.92	1.66	33.07	916.58	53.62

### Numerical method

#### Mathematical model

When studying solute transport in non-penetrating fractured media, it is necessary to consider groundwater flow and pollutant transport, and it is necessary to couple the groundwater flow model with the solute transport model.

1. *Model of groundwater flow* Groundwater flow is a very complex process, the fracture can generally be regarded as a water-conducting channel composed of two water-retaining surfaces, and has the characteristics of two-dimensional water flow movement. In non-penetrating fracture, groundwater flow only in the interconnected part of the region. Non-penetrating fractured groundwater flow is the transient groundwater flow in a heterogeneous anisotropic medium, which can be calculated and simulated using the Boussinesq Eq. (1)^55^:
  1 $$\left\{ {\begin{array}{*{20}l} {\mu \frac{\partial h}{{\partial t}} = \frac{\partial }{\partial x}\left( {K_{x} \frac{\partial h}{{\partial x}}} \right) + \frac{\partial }{\partial y}\left( {K_{y} \frac{\partial h}{{\partial y}}} \right) + \frac{\partial }{\partial z}\left( {K_{z} \frac{\partial h}{{\partial z}}} \right) + W,\;x,y,z \in \Omega ,\;t \ge 0} \hfill \\ {\left. {h\left( {x,y,z,t} \right)} \right|_{t = 0} = h_{0} ,\;x,y,z \in \Omega } \hfill \\ {\left. {h\left( {x,y,z,t} \right)} \right|_{{\Gamma_{1} }} = h_{1} \left( {x,y,z,t} \right),\;x,y,z \in \Gamma_{1} ,\;t \ge 0} \hfill \\ {\left. {K_{n} \frac{\partial h}{{\partial \overrightarrow {n} }}} \right|_{{\Gamma_{2} }} = q\left( {x,y,z,t} \right),\;\;x,y,z \in \Gamma_{2} ,\;t \ge 0} \hfill \\ \end{array} } \right.,$$
  where Ω is domain of fractured media; *x*, *y* and *z* are the space coordinates; *K*_*x*_, *K*_*y*_, *K*_*z*_ are the hydraulic conductivities along coordinate three axes; *K*_*n*_ is the hydraulic conductivity in normal direction of boundary surface; *t* is time; *n* is the normal direction of boundary surface; $\Gamma_{1}$ and $\Gamma_{2}$ is domain of the first and second type boundary, respectively; *h*_0_ is the initial groundwater table; *h*_*f*_ is the hydraulic head in the fractured system; *h*_1_ is the known groundwater table in the first type boundary; *μ* is the specific yield for unconfined aquifer and specific storage for confined aquifer; *q* is the unit area flux of the second type boundary, its value is positive for groundwater inflow and negative for outflow; W is the sinks and sources, such as the evaporation, precipitation and pumping rate.
2. *Model of solute transport* The solute transport process is the result of a combination of factors. The study of solute transport in non-penetrating fractured media needs to consider the adsorption of solute, and the transport of solute in groundwater conforms to Fick’s law. The contaminant control equation can be expressed as:
  2 $$\left\{ {\begin{array}{*{20}l} {R\theta \frac{\partial C}{{\partial t}} = \frac{\partial }{{\partial x_{i} }}\left( {\theta D_{ij} \frac{\partial C}{{\partial x_{j} }}} \right) - \frac{\partial }{{\partial x_{i} }}\left( {\theta v_{i} C} \right) - WC_{s} - WC - \lambda_{1} \theta C - \lambda_{1} p_{b} \overline{C}} \hfill \\ {C\left( {x,y,z,t} \right) = C_{0} \left( {x,y,z} \right) \left( {x,y,z} \right) \in \Omega ,\;t\; = \;0} \hfill \\ {\left. {C\left( {x,y,z,t} \right)} \right|_{{\Gamma_{1} }} = C\left( {x,y,z,t} \right) \left( {x,y,z} \right) \in \Gamma_{1} ,\;t \ge 0} \hfill \\ {\left. {\theta D_{ij} \frac{\partial C}{{\partial x_{j} }}} \right|_{{\Gamma_{2} }} = f_{i} \left( {x,y,z,t} \right)\begin{array}{*{20}c} {} & {} & {\left( {x,y,z} \right)} \\ \end{array} \in \Gamma_{2} ,\;t \ge 0} \hfill \\ \end{array} } \right.,$$
  where *θ* is the medium porosity, dimensionless; *t* is the time; *R* is the hysteresis coefficient, dimensionless; *C*_0_ (*x*,*y*,*z*) is the known concentration distribution; ${\overline{\text{C}}}$ is the solute concentration adsorbed by the medium skeleton; *p*_*b*_ is the medium density; *C* is the component concentration; *C*_*s*_ is the concentration of the component; *v*_*i*_ is the groundwater percolation velocity tensor; *D*_*ij*_ is the hydrodynamic dispersion coefficient tensor; *λ*_1_ is the primary reaction rate of the dissolved phase; *λ*_2_ is the reaction rate of the adsorbed phase; W is the source-sink term of water flow; *C*(*x*,*y*,*z*,*t*) is the concentration distribution on the fixed concentration boundary; Ω is the model simulation area; *f*_*i*_ (*x*,*y*,*z*,*t*) is the known dispersion flux function on the boundary; ${\Gamma }_{{1}}$ is the given concentration boundary; ${\Gamma }_{{2}}$ is the flux boundary.

#### Feflow software

Feflow (finite element subsurface flow system) was developed by the WASY Water Resources Planning and Systems Institute of WASY in Germany in 1979 and is one of the most complete groundwater numerical simulation software packages. Feflow adopts the Galerkin finite element method when simulating two-dimensional or three-dimensional stable or unsteady flow and the transport of contaminant, and can perform particle tracking and streamline simulation; simulation of contaminant, water flow field and temperature field can be carried out simultaneously. Feflow is equipped with several advanced numerical solution methods to control and optimize the solution process, which can solve quickly and directly, reduce numerical dispersion, and automatically adjust the simulation time step.

#### Parameter calibration method

The least-squares method is a commonly used curve fitting method with which hydraulic conductivity and dispersion coefficient can be corrected. The transient flow and solute transport functions obtained by the least-squares method can be expressed as:

3 $${\text{LSM}} = \sum\limits_{j = 1}^{m} {\sum\limits_{i = 1}^{n} {w_{i,j} \left[ {({\text{X}}_{O} - {\text{X}}_{c} )_{i,j} } \right]^{2} } } ,$$

where *n* is the number of given hydraulic heads and solute concentration; *m* is the number of time steps; *X*_*O*_ and *X*_*C*_ are the observed and calculated groundwater tables and solute concentration, respectively; *w*_*i*,*j*_ are weighting factors, which range from 0 to 1. Its value depends on location of observation holes. For high groundwater tables and solute concentration, a small weight is assigned; otherwise, the weight has a large value. When the value of LSM is less than a given predetermined error, the calibrated parameters are considered to be their optimal values.

## Results

### Experimental results of solute transport in fractured clay

According to the test results, the solute concentration of each observation point under different time conditions was obtained (Table 3). The measured values of solute concentrations at Obs1 and Obs2 increased slowly with time, while the measured concentrations of Obs3 and Obs4 increased relatively quickly with time. Due to the existence of the fracture, the solute breaks through the non-penetrating zone in the clay under the condition of hydraulic head pressure, and then the solute passes through the coarse sand and gravel layers rapidly. In the process of measuring the concentrations of Obs3 and Obs4, the value fluctuated to some extent, which may be related to the solute passing through the coarse sand and gravel layers at the bottom of model device.

**Table 3:** Relationship between solute concentration and measured time.

Measured time (days)		0	5	10	30	50	60
Solute concentration (g/L)	Obs1	0.63	8.56	17.52	30.52	44.92	45.78
	Obs2	0.53	4.91	14.12	26.47	41.01	42.22
	Obs3	0.51	15.29	25.31	41.91	48.63	48.95
	Obs4	0.61	20.20	25.92	44.85	49.56	50.62

The concentrations of all measured holes eventually stabilized. Obs3 and Obs4 reach a steady state in a relatively short time relative to Obs1 and Obs2, and Obs2 has the slowest increase. This shows that the existence of fracture leads to the phenomenon of preferential flow of solute in the clay. The solute quickly reaches the non-penetrating zone in the clay through the preferential flow. Although it is blocked by the non-penetrating zone, it will also preferentially reach in the bottom of the clay layer before its surrounding clay layer, which results in contamination of soil or aquifer below the clay impermeable layer. Therefore, in the case of using clay as an anti-seepage system in an engineering project, the fractures caused by the clay itself or construction actions have a certain impact on the safety of the engineering project.

The solute concentration also showed a certain variation in space. The distances of Obs1–Obs4 to the top of the clay layer were 7 cm, 17 cm, 28 cm and 52 cm, respectively. Before the start of the test, the solute concentrations of four holes were basically the same, which can be considered as background values (Table 3). With the increase of time, after 10 days, the solute concentrations of the four observation holes were 17.52 g/L, 14.12 g/L, 25.31 g/L and 25.92 g/L, respectively. The solute concentration of Obs2 is the smallest, and the solute concentration of Obs4 is the largest. The concentrations of Obs3 and Obs4 that were farther from the top of the model device were higher than those of Obs1 and Obs2 that were closer. The solute migrates along the fracture in the clay and quickly reaches the top of the non-penetrating zone in the clay. Although there is a non-penetrating zone, the solute quickly breaks down the non-penetrating zone with a high hydraulic water head pressure, which reduces the seepage resistance of the clay.

### Numerical results of solute transport in fractured clay

#### Parameter inversion analysis

**Figure 4:**
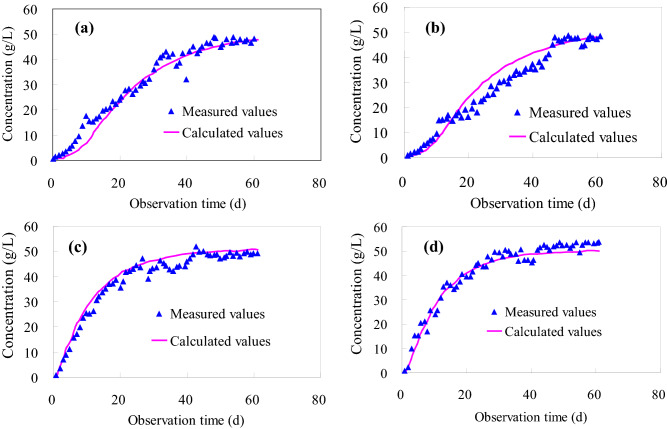
Comparison of experimental and numerical simulation results. (**a**) Obs1, (**b**) Obs2, (**c**) Obs3, (**d**) Obs4.

**Figure 5:**
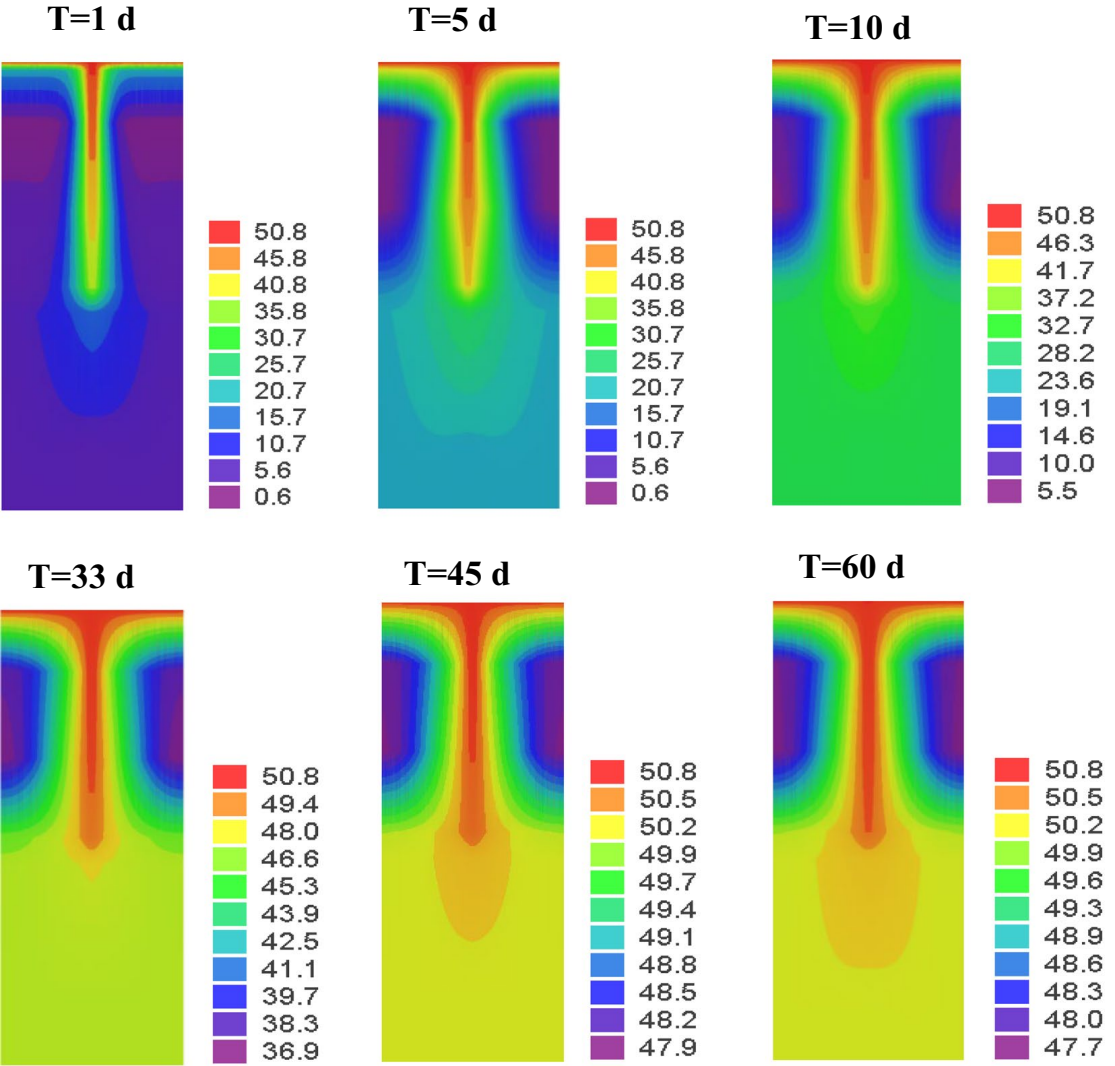
Cloud map of solute transport.

A 3D finite element model was established by Feflow software. The number of discrete nodes in the model was 38,240, the number of elements was 59,738, and the initial concentration of solute was 50.80 g/L. The numerical model conditions are consistent with the experimental model, and the hydraulic conductivity and dispersion parameters are adjusted according to the experimental observation data. So, the errors between the simulated and the experimental values are fitted (Fig. 4). It can be seen from Fig. 4 that the calculated value using the numerical model first increases and then tends to be stable, and the calculated value agrees well with the experimental value. The parameter inversion results are shown in Table 4. It can be seen from Fig. 5 that the solute quickly reached the bottom of the clay along the fracture after 1 day, but the diffusion of the solute in the clay basically did not change. After 5 days of solute migration, the solute gradually passed through the non-penetrating clay and reached the bottom of soil layer. After 60 days of solute transport, the solute concentration in all measured holes basically reached the initial value.

**Table 4:** Parameter inversion results.

Media types		Clay (m/s)	Fracture (m/s)	Corase sand (m/d)	Gravel (m/day)
Hydraulic conductivity		1 × 10^−6^	10.0	10.0	50.0
Dispersion	Longitudinal dispersion	0.01	25.0	10.0	10.0
	Lateral dispersion	0.005	5	1.5	4.5

#### Prediction of solute transport in fractured clay using the numerical method

**Figure 6:**
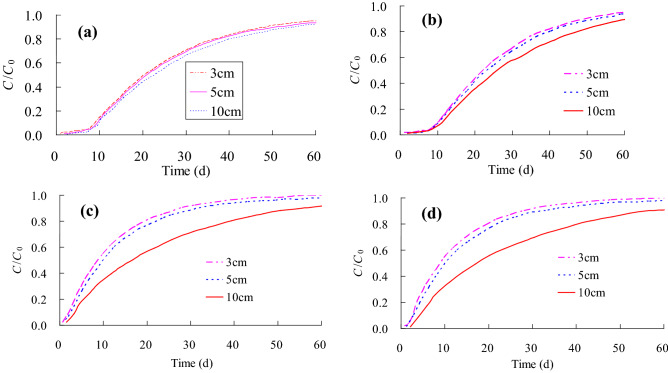
Concentration breakthrough curves for different thicknesses of the non-penetrating zone: (**a**) Obs1, (**b**) Obs2, (**c**) Obs3, (**d**) Obs4.

1. *Influence of non-penetrating zone thickness in the clay on solute transport* Under the experimental conditions, the thickness of the non-penetrating zone was 3 cm, and the length of the fracture was 25 cm. For the numerical simulation, the thickness of the non-penetrating zone is set as 5 cm and 10 cm, respectively. The simulation results are shown in Fig. 6. It can be seen from Fig. 6 that the calculated concentration values of Obs1 are not much difference for the non-penetrating zone of 3 cm, 5 cm and 10 cm. The solute concentrations after 10 days were 6.65 g/L, 6.31 g/L and 6.30 g/L, respectively. The values after 30 days were 35.35 g/L, 34.72 g/L and 33.96 g/L, respectively. With the increase of simulation time, the observation concentration basically tends to be stable. For Obs2, when the non-penetrating zone is 3 cm, 5 cm and 10 cm, the solute concentrations after 10 days are 6.19 g/L, 6.20 g/L and 5.53 g/L, respectively, and after 40 days the values are 41.91 g/L, 41.91 g/L, and 41.55 g/L. After 60 days, the values were 48.08 g/L, 47.93 g/L and 45.33 g/L, respectively. The solute concentration of the 10 cm thickness is relatively small comparing to the non-penetrating zone of 3 cm and 5 cm. The calculated results of solute concentration at Obs3 and Obs4 are basically consistent, which may be due to the instantaneous solute diffusion to the entire sand and gravel area when it reaches the sand layer after passing through the non-penetrating zone. Among them, the non-penetrating clay with a thickness of 10 cm has the best effect on preventing the migration of solute, where the solute concentration increases very slowly.
  The calculation results show during the process of solute transport in non-penetrating fractures, the thickness of non-penetrating zone has a great influence on the change of solute concentration at the bottom of non-penetrating zone. The measured holes that are farther from the fracture bottom are less affected by the thickness variation of the non-penetrating zone. Therefore, if there are non-penetrating fractures in the clay, the scale of the fracture has a direct and important impact on the migration and leakage of solute, and contaminants can easily migrate to groundwater through the clay with the non-penetrating fracture, which leads to groundwater pollution.
2. *Influence of hydraulic head height in the clay on solute* transport The solute transport is simulated with the height of hydraulic heads of 30 cm, 49 cm and 60 cm respectively. The calculation results are shown in Table 5. It can be seen from the table that with the increase of time, the solute concentration gradually increases, and the solute concentration increases rapidly at the beginning, and then gradually becomes stable, and finally reaches the initial concentration. Under the same time conditions, the higher the hydraulic head, the greater the solute concentration. Under the high head pressure, the time for the solute to penetrate the non-penetration zone will be shortened, and the solute will enter the groundwater at a faster rate. Therefore, for similar projects such as landfills, it is necessary to set up drainage measures at the bottom of the landfill to drain the leachate in time. If the leachate is too high at the bottom of the landfill, it is likely to break down the anti-seepage layer at the bottom of the landfill, which causes the landfill leachate to directly enter the groundwater. In addition, the thickness of the anti-seepage layer at the bottom of the landfill can also be increased, so that it is difficult for the leachate to penetrate the anti-seepage layer.
3. *Influence of fractured aperture in the clay on solute transport* Due to the irregularity and unpredictability of fractures in the clay, the fractures in clay are not only different in size, but also in width. The solute transport is considered when the fracture aperture is 1 mm, 5 mm and 10 mm, respectively. The calculation results of the solute concentration are shown in Table 6. It can be seen from the table that when the observation time is the same, the larger the fractured aperture is, the higher the solute concentration is, but it does not increase linearly. Under the condition of the same fractured aperture, the solute concentration gradually increases with time and tends to be stable after 60 days. Owing to the affection of the fracture, the solute concentration at Obs3 and Obs4 increases rapidly and increases with the increase of the fractured aperture. This paper mainly discusses the influence of one fracture. In actual projects, due to construction and management reasons, there may be multiple fractures, which greatly increases the risk of pollutants migrating to groundwater.

**Table 5:** Relationship of solute concentration and height of hydraulic head.

Number of observation holes	Time (days)	Height of hydraulic head (cm)		
		30	49	60
Obs1	10	5.51	6.31	6.63
	30	32.92	34.72	34.45
	60	46.67	47.93	48.31
Obs2	10	2.76	3.90	4.62
	30	28.14	33.41	35.20
	60	45.43	47.93	48.47
Obs3	10	20.11	29.45	32.24
	30	39.82	46.56	47.74
	60	48.35	50.26	50.49
Obs4	10	17.57	27.01	30.00
	30	39.63	46.56	47.81
	60	48.32	50.28	50.48

**Table 6:** Relationship of solute concentration and fractured aperture.

Number of observation holes	Time (days)	Fractured aperture (mm)		
		1.0	5.0	10.0
Obs1	10	5.36	6.31	7.87
	30	33.19	35.56	38.50
	60	46.05	47.93	49.12
Obs2	10	3.65	5.53	9.01
	30	27.04	34.36	39.16
	60	43.76	47.92	49.35
Obs3	10	15.52	29.45	33.91
	30	34.58	46.53	48.37
	60	45.79	50.26	50.57
Obs4	10	14.67	29.01	33.81
	30	34.24	46.56	48.42
	60	45.67	50.28	50.58

## Discussion

Previous studies have attempted to investigate the influence of fracture characteristics on the migration of groundwater contaminants through indoor physical experiments and numerical simulation. The solute transport law of groundwater was also analyzed from the perspective of clay properties. Neretnieks et al.^42^ investigated the migration characteristics of solute in fractured clay using tracer tests. Seol et al.^48^ studied the two-dimensional parallel plate fracture-matrix system by a numerical method. Liu et al.^50^ used a numerical simulation method to study the characteristics of water flow in a two-dimensional fracture network model. Thousands of fractures were randomly generated in a large area. The simulation results showed that the direction of water flow is mostly vertical. There are few systematic studies on the effects of the non-penetrating zone thickness, the hydraulic head height and the fracture aperture on solute transport in non-penetrating fractured clay. The experiments were carried out using the self-developed experimental device and the simulation were carried out using the verified numerical model. The results showed that the increase in the hydraulic head pressure and the fracture aperture leaded to an increase in solute concentration, which was similar to the results of Reynolds et al.^13,50^. The results showed that the increase of the thickness of the non-penetrated zone had significantly improved the impermeability of the clay, and this result was consistent with the finding of Detwiler et al.^21^. The study of contaminants migration in non-penetrating fractured clay has enriched the theory of groundwater contaminant transport, it is of great significance to on-site projects such as landfills.

## Conclusions

When there are fractures in the clay and do not penetrate the clay layer, the solute will quickly pass through the fracture to reach a deeper position in the clay layer. Compared with the solute migration in the intact clay, there is a relatively preferential flow phenomenon. However, due to the existence of the clay non-penetrating zone, there is a relative barrier to the solute transport. Due to the thin layer of the non-penetrating zone, the effect of blocking solute migration is not obvious. After a period of time, the solute migrates through the non-penetrating zone and reaches the sand and gravel layers at the bottom of the clay. Solute will spread in the sand and gravel layers. The calibrated numerical model was used to simulate and predict the solute migration law when the conditions such as the non-penetrating zone thickness, the hydraulic head height and fracture aperture were changed. The variation of non-penetration zone thickness directly changed the effectiveness of clay impermeability. At the same time, the change of hydraulic head height and the change of fracture aperture also had some effect on the effectiveness of clay impermeability. The related research results can provide a theoretical basis for the management and remediation of contaminants in fractured clay. It can also provide guidance for avoiding groundwater pollution on a larger scale. The further research is needed to investigate the effects of different soil types, water flow conditions and contaminant characteristics on solute transport in non-penetrating fractures.

## Data Availability

All data generated or analysed during this study are included in this published article.
